# Analysis of Material-Characterization Properties of Post-Production Waste—The Case of Apple Pomace

**DOI:** 10.3390/ma15103532

**Published:** 2022-05-14

**Authors:** Weronika Tulej, Szymon Głowacki

**Affiliations:** Institute of Mechanical Engineering, Warsaw University of Life Sciences, 02-787 Warsaw, Poland; szymon_glowacki@sggw.edu.pl

**Keywords:** apple pomace, post-production waste, material characteristics, differential scanning calorimetry, thermogravimetry

## Abstract

The paper presents the material-characterization properties of apple pomace—the post-production waste of juice pressing. Tests were carried out on the basic physical properties of apple pomace: color, specific-density, and energy properties. Extensive material-composition analyses based on DSC (differential scanning calorimetry) and TGA (thermogravimetry) methods were also performed. It has been shown that pomace, due to its energy value, can be a good fuel. The obtained thermal data confirm the presence of cellulose, hemicelluloses, lignins and pectins in the analyzed pomace. The results confirm that dried apple pomace is microbiologically stable with good health-promoting properties.

## 1. Introduction

The apple is one of the oldest fruits known to mankind. It is one of the most important horticultural products in the world and is also one of the most sold fruits in the world [1,2]. Apples are the fruit most often grown in the European Union, and Poland ranks in the leading position [3]. Thanks to low costs and a large scale of production, Poland is the world leader in fresh apple exports and the second in the world (China—first place) in exports of apple-juice concentrate. For decades apples have been one of the most important fruits, both in terms of production and consumption in Poland. Apples are an important component of the fruit and vegetable diet; their consumption reached 14.20 kg per year per capita in 2016 [4,5].

Fruit and vegetable processing generates post-production waste. The amount of waste that is generated from fruit processing has been determined to be between 10 and even 35% by weight of processed raw material [6]. The largest share of the generated waste is pomace, which is the residue from the mechanical pressing of juice. The apple-processing industry provides about 20 million tons/year of waste worldwide [7]. Waste disposal is a significant problem; it poses a threat to the environment and human health. Adding value to the waste material will help solve this problem and increase the benefits. The primary goal of waste valorization is the transition to a circular economy and the mitigation of the hazardous effects of waste [8]. Pomace is a by-product of the processing of fruit into juices and drinks, cider and wine. In the juice-production process, the mass of the pomace depends on the pressing capacity. The pomace can be considered a mixture of pulp, peel, seeds and stems. Apple pomace is generated as ‘press-cake’ after juicing the fruit, via a series of steps which may include milling, primary mash enzymation (liquefaction), primary juice extraction, leaching, heating, secondary liquefaction, and secondary juice extraction [9]. Pomace should be treated as a half-finished product, which may be further processed, as it is rich in nutrients: protein, saccharides, mineral compounds, fiber, pectin, lipids, vitamins and organic acids [10]. It is a material for composting, an ingredient of feeds, a food product and a material for the production of fruit fiber, bio-oils, pectins, food colorings, natural dyes and polyphenol extracts [11,12].

Apple pomace can be a source of energy [13]. The pomace can be used to produce energy in the form of biogas (conversion of the pomace through an anaerobic treatment process) or biofuel (extraction of accumulated energy from biomass by fermentation of sugars into alcohol) [14,15]. Research results [16] show that apple pomace is an excellent raw material for the production of ethanol, which can be used as a biofuel or a drink, e.g., cider. Due to the variable composition and abundance of material, apple pomace can be a suitable substrate for use in biorefining practices where substrate conversion, energy and chemical consumption are optimized. The valorization of apple pomace as part of the biorefinery approach is more attractive and economically viable than its use as a single product source. The use of biomass waste in biorefineries has significant potential for the production of biofuels and organic fertilizers [17,18].

The problem of the rational management of apple waste is not a simple matter, and the way of dealing with it in a production plant depends on technical and organizational possibilities of the company. This waste is produced in a large amount and in a short time as a raw material for reuse [19]. Drying the pomace [20] is necessary to extend its consumption and processing life. The production of solid biofuels for direct combustion also requires drying [21].

Fruit pomace is a chemically and microbiologically unstable material. This material is currently used in many ways, including as a feed ingredient, composting feedstock, food product, and raw material for pectin, fruit fibers, bio-oils, natural dyes, or polyphenolic extracts [22].

The main purpose of the presented considerations is to define the basic material properties of apple pomace. This will facilitate the determination of the potential of the raw material and the influence of the drying temperature on the thermal characteristics of the pomace. Due to the wide spectrum of use of pomace—in food and feed raw materials, the biodegradable-packaging industry, and energy use—determining the basic physical and chemical properties (specific density, water activity, color, heat of combustion, antioxidant capacity, polyphenol content, thermal characteristics) can direct their use.

## 2. Materials and Methods

Pomace from apple-juice production was used for this study. Fresh apple pomace obtained from Energreen, Poland was used for the research. The pomace was collected immediately after squeezing the juice from various apple varieties at the end of September. Fresh pomace dried at 60 °C by natural convection (drying was continued until the equilibrium water content of the dried material was reached) and dried to dryness at 105 °C were considered. The moisture content of the fresh material oscillated around 75%. The drying temperature of 60 °C was chosen as the optimum from among the other drying temperatures, the results of which are presented in the article [11].

▪ Determination of the energy value of apple pomace: Measurement by sample-preparation bomb. A calorimeter (sample-preparation bomb) KL-12 Mn made by PRECYZJA-BIT was used. The device allows for measuring of the heat of combustion of solids. It is made up of a container into which the test sample is placed and filled with oxygen and then brought to ignition. The heat of combustion at constant volume is determined from the increase in temperature [23].
▪ Determination of the mass loss of a substance depending on the temperature: Samples were analyzed using a Discovery TGA (TA Instruments, Newcastle, DE, USA) thermogravimetric analyzer. Measurements were performed in a nitrogen atmosphere at a gas-flow rate of 25 mL/min. The samples were placed on platinum dishes. The weight of the samples ranged from 7–8 mg. Measurements were performed in the temperature range of 50–1000 °C with a heating rate of 10 °C/min. Based on the TG curves (black) showing the dependence of mass on temperature, the first derivative (DTG) was obtained.
▪ Determination of the mass loss of a substance depending on the temperature: Samples were analyzed using a Discovery TGA (TA Instruments) thermogravimetric analyzer. Measurements were performed in an oxygen atmosphere at a gas-flow rate of 25 mL/min. The samples were placed on platinum dishes. The weight of the samples ranged from 7–8 mg. Measurements were performed in the temperature range of 50–1000 °C with a heating rate of 10 °C/min. Based on the TG curves (black) showing the dependence of mass on temperature, the first derivative (DTG) was obtained.
▪ Thermal characteristics of apple pomace: Measurement with DSC (Differential Scanning Calorimeter—DSC Q200 by TA Instruments). Thermal analysis in special aluminum dishes using nitrogen, temperature range 35 °C to 500 °C, heating rate 5 °C/minute, nitrogen flow rate 50 mL/min.
▪ Thermal characteristics of apple pomace: Measurement using a PDSC (differential pressure scanning calorimeter—PDSC Q20 by TA Instruments). Thermal analysis in special aluminum dishes in the presence of oxygen under atmospheric pressure, temperature range 35 °C to 500 °C, heating rate 5 °C/minute, oxygen flow rate 50 mL/min.
▪ Determination of transformation enthalpies for apple pomace: Measurement using the DSC Q200 by TA Instruments or the PDSC Q20 by TA Instruments. Thermal analysis in special aluminum dishes using nitrogen or oxygen, temperature range 35 °C to 500 °C, heating rate 5 °C/minute. The resulting curves were analyzed using TA Universal Analysis 2000 software (TA Instruments, Newcastle, DE, USA).
▪ Color analysis of apple pomace: The color of samples was measured five times using the Minolta Chroma Meter CR-400 (Minolta Co., Ltd., Osaka, Japan). The results were obtained with reference to the International Commission on Illumination (CIE) L* a* b* color space, where L* stands for brightness, a* values vary between negative (green) and positive (red), and b* values vary between negative values labeled as blue color and positive values labeled as yellow shades [24,25]. The total color change of the dried material was expressed as DE*. It is equal to the square root of the sum of the squares of the differences of each of the three coordinates of the two colors. Such a distance, denoting the color difference, is determined from the Formula (1):
  (1) $$\Delta E\;=\sqrt{{\left(\Delta L\right)}^{2}+{\left(\Delta a\right)}^{2}+\left(\Delta b{)}^{2}\right)},$$
▪ Determination of the true density of pomace: The true density (ρT) was determined by calculating the ratio of the dry mass (m) to the total volume (Vs) of the sample, excluding air pores according to the Equation (2) and expressed in kg m^−3^:
  (2) ρT =mVs,

The material was powdered and weighed using an analytical balance accurate to 0.0001 g (XA 60/220/X Radwag, Radom, Poland), and the air-free total volume (Vs) was measured using a HumiPyc™/model 2 Gas Pycnometer (InstruQuest Inc., Coconut Creek, FL, USA) [26].

▪ Water activity for apple pomace: Water activity measures the degree of water removal and the amount of residual moisture of the dried products [27]. In order to eliminate the possibility of microbial growth and adverse enzymatic activity, dried products should have a low water-activity index of 0.60 to 0.80. Spoilage-causing bacteria are inhibited at a water-activity index of 0.91, and most mold types are inhibited at an index of 0.80 [28]. However, for dried leaves or tea products, the suggested index should be below 0.267 [29]. Water activity was measured three times using an AquaLab DewPoint 4Te water-activity meter (Decagon Devices Inc., Pullman, WA, USA). Results were expressed as means. Water activity was measured at a mean temperature of 24.9 ± 0.05 °C.
▪ Trolox equivalent antioxidant capacity (TEAC) and ferric reducing antioxidant potential (FRAP): The solvent for analysis was prepared as previously described by Wojdylo et al. [30]. The ABTS+ activity of the sample was determined according to the method of Re et al. [31]. The total antioxidant potential of the sample was determined using the ferric-reducing-antioxidant-potential (FRAP) assay by Benzie and Strain [32] as a measure of antioxidant power. All determinations were performed in triplicate using a Shimadzu UV-2401 PC spectrophotometer (Kyoto, Japan). Test results were expressed in terms of micromoles of Trolox per gram of dry weight.
▪ Analysis of total phenolic content: The total phenolic content was determined by the Folin–Ciocalteu method [33]. The pomace extract (0.1 mL) was mixed with 2 mL of water and 0.2 mL of Folin–Ciocalteu reagent. The mixture was incubated at room temperature for 3 min and 1 mL of 20% sodium carbonate was added. Absorbance was measured using a UV–vis spectrophotometer (Shimadzu, UV-2401 PC, Kyoto, Japan) at 765 nm after 1 h incubation at room temperature. The quantification was determined by the standard curve of gallic acid. Gallic acid is a standard substance that provides a quantitative reference to polyphenols. Results were expressed as gallic-acid equivalents (in milligrams per 100 g sm). Polyphenol extracts were prepared as previously described [34]. The presence of polyphenols was identified using the Acquity Ultraperformance LC system. Data obtained from LC-MS were evaluated in MassLynx 4.0 ChromaLynx Application Manager software (Waters Corporation, Milford, MA, USA).

## 3. Results

Considering the results obtained, it can be concluded that apple pomace is characterized by a rather high heat of combustion of about 20 kJ/g (Table 1). For comparison, the heat of combustion of apricot kernels is almost 22 kJ/g [35], straw is about 18 kJ/g [36], wood is 16–21 kJ/g, and peat is 16 kJ/g [37].

**Figure 1:**
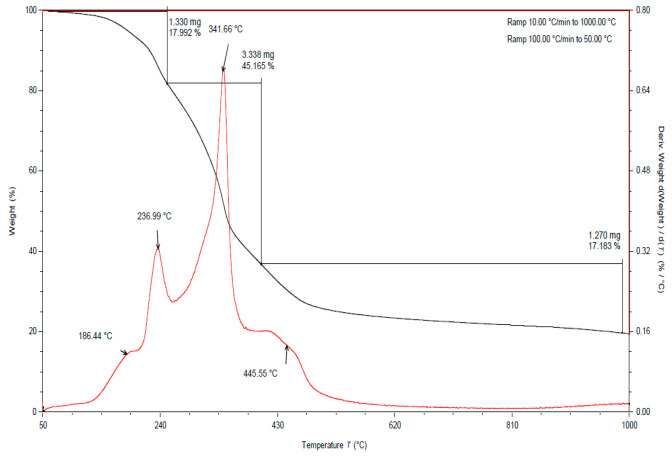
TGA curve for apple pomace dried at 60 °C. Analysis under nitrogen atmosphere (black line is a curve of mass loss versus temperature, red line is the first derivative).

**Figure 2:**
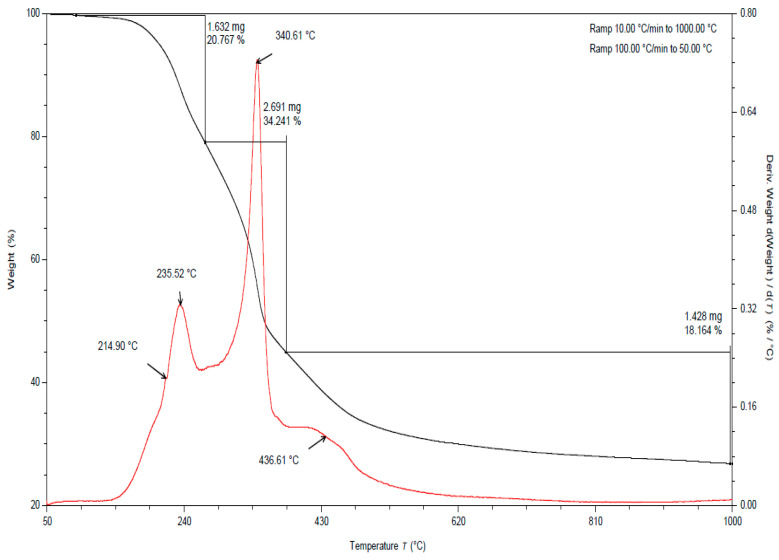
TGA curve for apple pomace dried to dry weight. Analysis in a nitrogen atmosphere (black line is a plot of mass loss versus temperature, red line is the first derivative).

**Figure 3:**
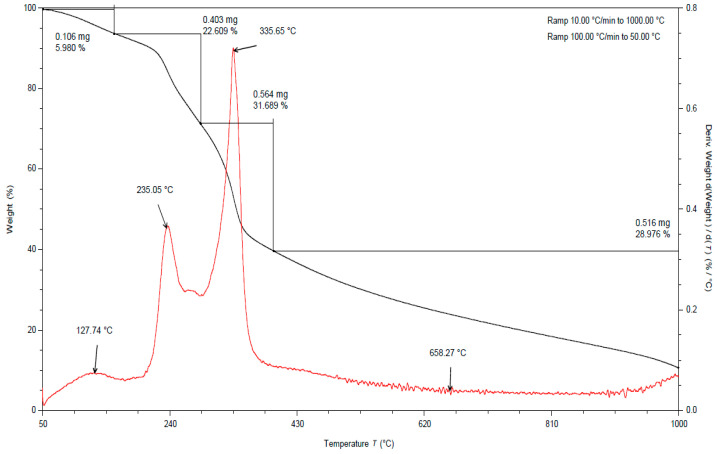
TGA curve for fresh apple pomace. Analysis in a nitrogen atmosphere (black line is a plot of mass loss versus temperature, red line is the first derivative).

In all the TGA curves (Figure 1, Figure 2 and Figure 3) obtained for the tested pomace, the highest percentage of weight loss related to cellulose degradation was observed (from 32 to 45% in the case of the analysis in the presence of nitrogen). Indeed, in this temperature range there is a peak characteristic of cellulose, with a maximum between 336 and 342 °C when analyzed in the presence of nitrogen. According to the literature data, thermal decomposition of cellulose occurs at temperatures between 315 and 400 [38].

By analyzing the TGA curves obtained for the tested pomace, a quite distinct percentage of weight loss was also observed, related to the degradation of hemicelluloses and pectins. Indeed, in this temperature range there are peaks characteristic of pectins and hemicelluloses with maxima ranging from 186 to 215 °C and 235 to 237 °C, respectively, when analyzed in the presence of nitrogen. The weight loss in this temperature range ranged from 18 to 23% (Table 2). For fresh pomace, only one peak was observed in this temperature range with a maximum of 235 °C.

In the case of fresh pomace, the analysis of TGA curves (analysis in the presence of nitrogen) allowed for the identification of an additional peak with a maximum of about 128 °C (Figure 3). Since this is a material that contains significant amounts of water, it can be assumed that these peaks were related to the loss of adsorbed and structural water. The DSC curves obtained for the samples tested under nitrogen atmosphere also showed endothermic peaks with maxima ranging from 120 to 155 °C (Table 3). The enthalpy for the transformation occurring in this temperature range for fresh pomace was about 40 times higher than for pomace dried to dry weight, so this peak is characteristic of water (Table 4).

By analyzing the TGA curves (Figure 1, Figure 2 and Figure 3) obtained for the tested pomace, a significant percentage of weight loss was also observed (from 17 to 29% in the case of nitrogen analysis) at temperatures above 400 °C. The peaks in this temperature range are indistinct. The maxima for these peaks ranged from 437 to 658 °C (measured in the presence of nitrogen). Thermal decomposition of lignins can occur in this temperature range. The literature data report that lignin degradation occurs over a wide temperature range (280 to 500 °C) [38].

**Figure 4:**
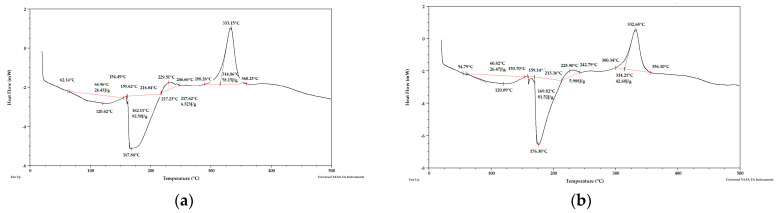
DSC curves for apple pomace dried at 60 °C (**a**) sample 1, (**b**) sample 2. Analysis under nitrogen atmosphere.

**Figure 5:**
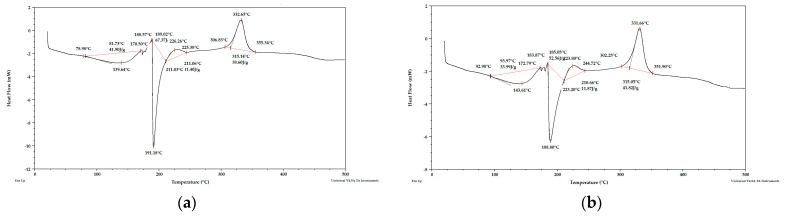
DSC curves for apple pomace dried to dry weight (**a**) sample 1, (**b**) sample 2. Analysis under nitrogen atmosphere.

**Figure 6:**
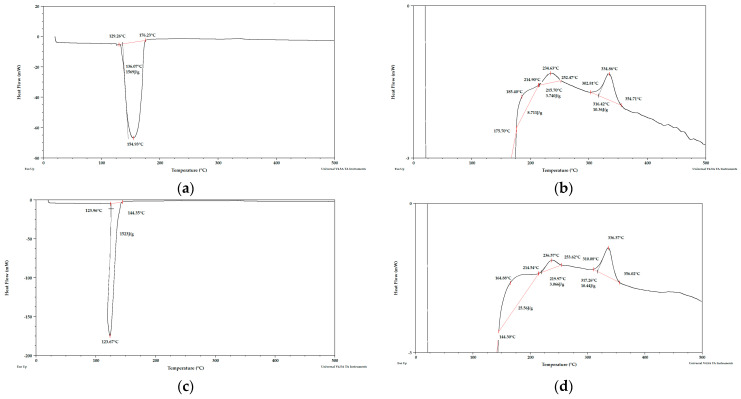
DSC curves for fresh apple pomace (**a**) sample 1, (**b**) sample 1 enlarged, (**c**) sample 2, (**d**) sample 2 enlarged. Analysis under nitrogen atmosphere.

Taking into account the literature data and analyzing the DSC curves (Figure 4, Figure 5 and Figure 6) it can be concluded that there was a significant amount of cellulose in the analyzed apple pomace. The DSC curves were characterized by the presence of an exothermic peak that is characteristic of cellulose with a maximum between 331 and 336 °C when analyzed in the presence of nitrogen. According to the literature data, thermal decomposition of cellulose occurs at temperatures between 315 and 400 °C [38].

Considering the DSC curves obtained for the analyzed samples, it can also be concluded that all the curves showed an exothermic peak with a maximum between 224 and 237 °C (Table 2) when analyzed in the presence of nitrogen. This temperature range is characteristic of both pectins and hemicelluloses. The literature data report that degradation of hemicelluloses occurs between 200 and 260 °C [38] and pectins between 180 and 270 °C [39].

The DSC curves obtained for the samples tested under nitrogen atmosphere also showed endothermic peaks with maxima between 120 and 155 °C. The enthalpy for the transformation occurring in this temperature range for fresh pomace was about 40 times higher than for pomace dried to dry weight, so this peak is characteristic of water. Considering the percentage residue of substances that did not degrade at temperatures up to 1000 °C, it can be concluded that the analyzed apple pomace may contain quite a lot of mineral components (residues at 1000 °C ranging from 11 to 26%; analysis under nitrogen atmosphere).

**Figure 7:**
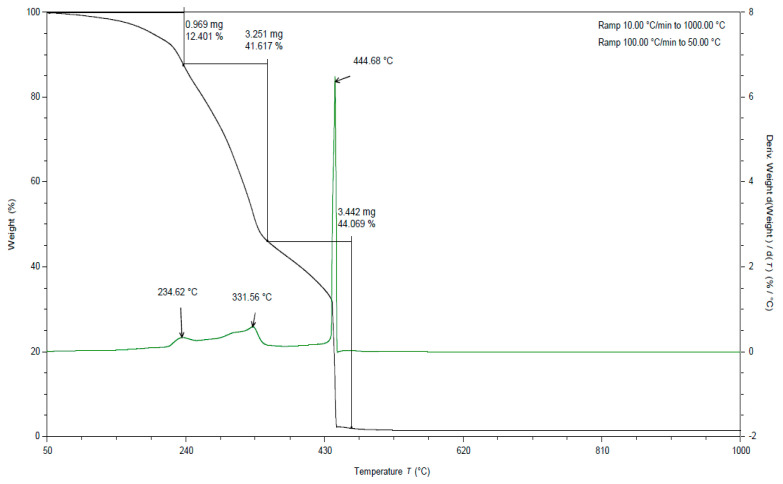
TGA curve for apple pomace dried at 60 °C. Analysis under oxygen atmosphere (black line is a curve of mass loss versus temperature, green line is the first derivative).

**Figure 8:**
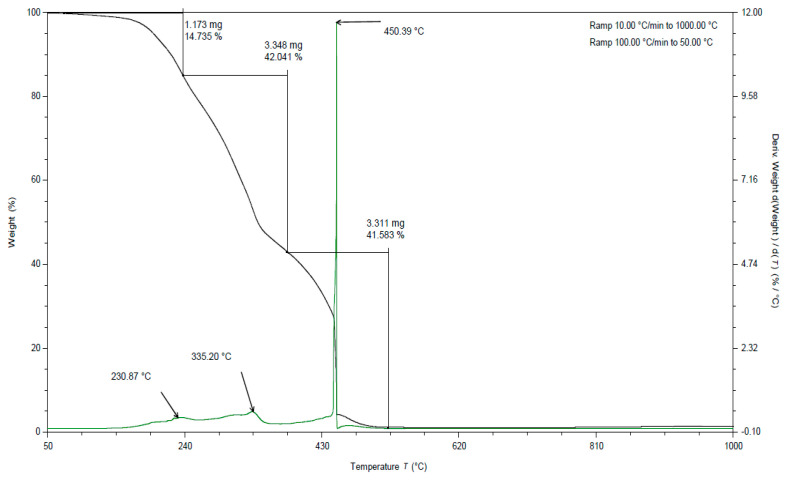
TGA curves for apple pomace dried to dry weight. Analysis in an oxygen atmosphere (black line is a curve of mass loss versus temperature, green line is the first derivative).

**Figure 9:**
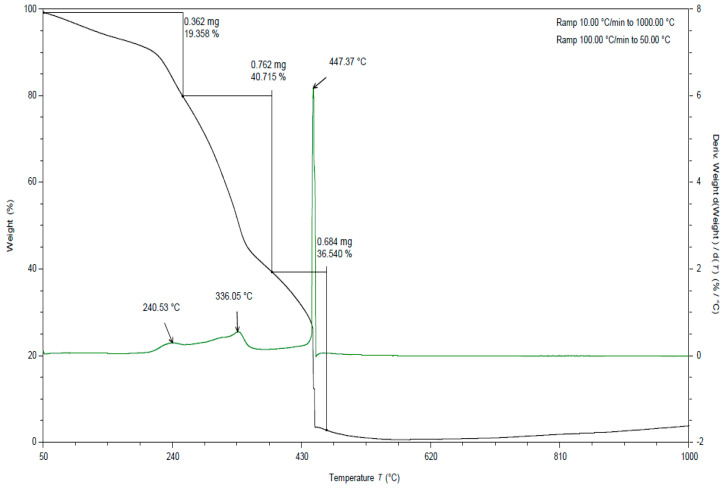
TGA curves for fresh apple pomace. Analysis in an oxygen atmosphere (black line is a curve of mass loss versus temperature, green line is the first derivative).

In all TGA curves (Figure 7, Figure 8 and Figure 9) obtained for the tested pomace, the highest percentage of weight loss related to cellulose degradation was observed (from 41 to 42% in the case of the analysis in the presence of oxygen). Indeed, in this temperature range there is a peak characteristic of cellulose with a maximum between 332 and 336 °C when analyzed in the presence of oxygen (Table 5).

When analyzing the TGA curves obtained for the tested pomace, a significant percentage of weight loss was also observed (from 37 to 44% in the case of analysis in the presence of oxygen) at a temperature above 400 °C (Table 5). The peaks occurring in this temperature range are indistinct. The maxima for these peaks ranged from 445 to 450 °C (measured in the presence of oxygen). Thermal decomposition of lignins can occur in this temperature range. The literature data report that lignin degradation occurs over a wide temperature range (280 to 500 °C) [38]. Exothermic peaks occurring at temperatures above 400 °C can be observed for DSC curves obtained for samples analyzed in an oxygen atmosphere (peak maximum from 441 to 472 °C).

**Figure 10:**
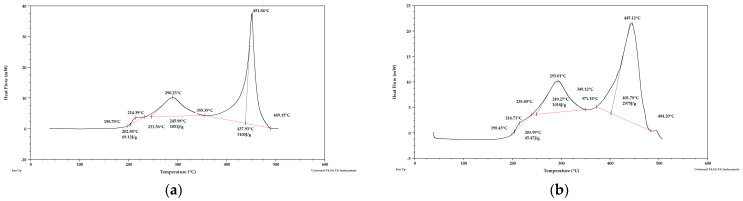
PDSC curves for apple pomace dried at 60 °C (**a**) sample 1, (**b**) sample 2. Analysis under oxygen atmosphere.

**Figure 11:**
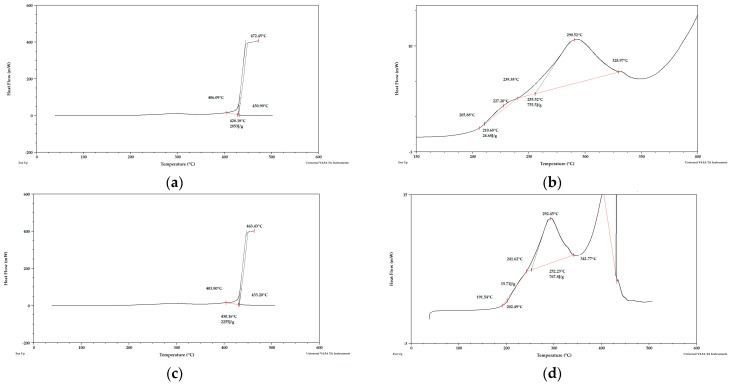
PDSC curves for apple pomace dried to dry weight (**a**) sample 1, (**b**) sample 1 enlarged, (**c**) sample 2, (**d**) sample 2 enlarged. Analysis in an oxygen atmosphere.

**Figure 12:**
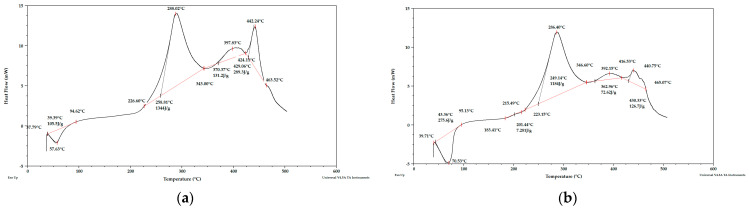
PDSC curves for fresh apple pomace (**a**) sample 1, (**b**) sample 2. Analysis in an oxygen atmosphere.

Taking into account the literature data and analyzing the DSC curves (Figure 10, Figure 11 and Figure 12) it can be concluded that there was a significant amount of cellulose in the analyzed apple pomace. The DSC curves are characterized by the presence of an exothermic peak that is characteristic of cellulose with a maximum between 290 and 397 °C when analyzed in the presence of oxygen. According to the literature data, thermal decomposition of cellulose occurs at temperatures between 315 and 400 °C.

The DSC curves (analysis in the presence of oxygen) obtained for fresh apple pomace (Figure 12) showed an endothermic peak with a maximum between 58 and 71 °C (Table 6). No endothermic peak was observed for pomace dried at 60 °C and dried to dry weight. (Table 7). Since this is a material that contains significant amounts of water, it can be assumed that these peaks were related to the loss of adsorbed and structural water.

Color is one of the most important parameters that determine the quality of raw materials and processed products [40]. This characteristic affects consumer acceptance of the product [41].

Color characteristics of apple pomace were given in CIE L* a* b* units. The color values of convection-dried apple pomace are shown in Table 8. The tested parameters include brightness (L*), 0 (black) to 100 (white); greenness or redness (a*), −60 (green) to 60 (red); and blueness or yellowness (b*), −60 (blue) to 60 (yellow). Color changes in biological materials subjected to drying are usually due to a loss of green pigments and carotenoids or enzymatic browning [42]. All color values are given in Table 8.

The true density (Table 9) of the sample was calculated as the mass-to-volume ratio of the ground sample excluding air pores. Low temperature or supporting drying with microwaves causes the smallest change in the true density of the dried material.

For comparison, the true density of energy crops: Virginia fanpetals—1150.5 kg/m^3^; multiflora—1117.1; giant miscanthus—779.9 [43].

Water activity is an indicator of the relative humidity of the atmosphere closest to the sample under test. It determines the course of biological processes and especially affects the growth of microorganisms. Water activity in foods indicates the degree to which water molecules are associated with food ingredients. Its value indicates the availability of water to microorganisms and thus the possibility of their growth, which affects product quality and shelf life [44]. In general, microbiologically safe material has a water activity of less than 0.6. The reduction in water activity due to drying minimizes the decrease in food quality and microbial activity [45]. For apple pomace dried by convection at 60 °C, this index is 0.1845, so we can see that dried apple pomace is microbiologically safe.

Polyphenols are natural compounds exclusively synthesized by plants that exhibit bioactivity to modulate oxidative and inflammatory stress, alter macronutrient digestion, and exert prebiotic-like effects on intestinal microflora [46]. The total polyphenol content of convection-dried apple pomace at 60 °C was 750.06 mg gallic acid/100 g (Table 10). The amount of extracted polyphenols depends on the extraction solvent, temperature, weight-to-solvent ratio and pre-treatment of the biomass prior to extraction. Solvent type and temperature affect the efficiency of TPC extraction [47,48].

The antioxidant activity of FRAP was 805.595 µmol/100 g product (Table 10). On the other hand, the ABTS (Trolox-equivalent antioxidant capacity) activity was 624.99 µmol/100 g product (Table 10). Leong and Shui [49] classified apples as a fruit with medium antioxidant capacity after testing a group of 27 fruit pulps.

## 4. Conclusions

Apple pomace is a fairly good material in terms of energy; it can be a substrate for the production of pellets and briquettes, as it has a relatively high energy value of about 20 MJ/kg. This value is one of the highest in relation to other materials of plant origin that are used for energy purposes. This is also confirmed by the true-density value.

Taking into account the literature data and analyzing the DSC, PDSC and TGA curves, it can be concluded that there was a significant amount of cellulose in the apple pomace analyzed. Considering the DSC curves obtained for the analyzed samples, it can also be concluded that all the curves showed an exothermic peak whose temperature range is characteristic of both pectins and hemicelluloses.

By analyzing the TGA curves obtained for the tested pomace, a significant percentage of weight loss was also observed at temperatures above 400 °C. The peaks in this temperature range are indistinct. Thermal decomposition of lignins can occur in this temperature range. The obtained thermal data confirmed the presence of cellulose, hemicelluloses, lignins and pectins in the analyzed pomace.

The results of the research of apple-pomace color indicated that it is a material with increased sensitivity to browning compared to other forms of dried apples. These differences amounted to over 30%. The determination of water activity allowed us to state that the dried apple pomace is microbiologically stable. In terms of bioactivity, apple pomace can be considered as a material with good health-promoting properties.

## Figures and Tables

**Table 1:** Heat of combustion of apple pomace.

Name	Pomace Dried at 60 °C			Pomace Dried to Dry Weight		
Combustion heat (J/g)	Measurement 1	Measurement 2	Average	Measurement 1	Measurement 2	Average
	19,343	19,991	19,667	20,684	20,216	20,450

**Table 2:** Percent weight loss and peak maximum temperature (°C) for apple-pomace samples based on the obtained TGA curves. Analysis under nitrogen atmosphere.

Name	Peak I	Peak II	% Weight Loss for Peaks I and II	Peak III	% Weight Loss for Peak III	Peak IV	% Weight Loss for Peak IV	Remains in 1000 °C
	Tmax	Tmax		Tmax		Tmax		
Fresh pomace	127.74	235.05	5.98 and 22.609	335.65	31.689	658.27	28.976	10.746
Pomace dried at 60 °C	186.44	236.99	17.992	341.66	45.165	445.55	17.183	19.66
Pomace dried to dry weight	214.90	235.52	20.767	340.61	34.241	436.61	18.164	26.828

**Table 3:** Peak maximum temperatures (°C) for apple-pomace samples based on the obtained DSC curves. Analysis under nitrogen atmosphere.

Name	Peak I		Peak II		Peak III		Peak IV	
	T1	T2	T1	T2	T1	T2	T1	T2
Fresh pomace	154.93	123.67	185.40	164.88	234.63	236.57	334.86	336.37
Pomace dried at 60 °C	120.62	120.09	167.84	176.30	229.51	225.90	333.15	332.68
Pomace dried to dry weight	139.64	143.61	191.18	188.80	226.26	223.80	332.65	331.66

**Table 4:** Enthalpies of transformation (J/g) for apple-pomace samples based on the obtained DSC curves. Analysis under nitrogen atmosphere.

Name	Peak I		Peak II		Peak III		Peak IV	
	ΔH1	ΔH2	ΔH1	ΔH2	ΔH1	ΔH2	ΔH1	ΔH2
Fresh pomace	1569	1523	8.711	25.56	3.74	3.07	10.36	10.44
Pomace dried at 60 °C.	24.43	26.47	92.50	81.52	6.323	5.988	58.15	42.68
Pomace dried to dry weight	41.90	33.99	67.37	52.56	11.40	11.87	38.60	41.82

**Table 5:** Percent weight loss and peak maximum temperature (°C) for apple-pomace samples based on the obtained TGA curves. Analysis in an oxygen atmosphere.

Name	Peak I	% Weight Loss for Peak I	Peak III	% Weight Loss for Peak III	Peak IV	% Weight Loss for Peak IV	Remains in 1000 °C
	Tmax		Tmax		Tmax		
Fresh pomace	240.53	19.358	336.05	40.715	447.37	36.540	3.387
Pomace dried at 60 °C	234.62	12.401	331.56	41.617	444.68	44.069	1.913
Pomace dried to dry weight	230.87	14.735	335.20	42.041	450.39	41.583	1.641

**Table 6:** Peak maximum temperatures (°C) for apple-pomace samples based on the obtained DSC curves. Analysis in an oxygen atmosphere.

Name	Peak I		Peak II		Peak III		Peak IV	
	T1	T2	T1	T2	T1	T2	T1	T2
Fresh pomace	57.63	70.53	288.02	286.40	397.83	392.15	442.24	440.75
Pomace dried at 60 °C.	-	-	214.39	214.71	290.23	293.01	451.84	445.12
Pomace dried to dry weight	-	-	227.20	202.49	290.52	292.45	472.45	463.43

**Table 7:** Enthalpies of transformation (J/g) for apple-pomace samples based on the obtained DSC curves. Analysis in an oxygen atmosphere.

Name	Peak I		Peak II		Peak III		Peak IV	
	ΔH1	ΔH2	ΔH1	ΔH2	ΔH1	ΔH2	ΔH1	ΔH2
Fresh pomace	105.50	275.60	1344	1184	131.20	72.62	289.30	126.70
Pomace dried at 60 °C.	-	-	69.13	45.47	1051	1018	3100	2975
Pomace dried to dry weight	-	-	24.68	15.71	755.5	767.8	2853	2255

**Table 8:** Color characteristics of apple pomace given in CIE L* a* b* units.

Sample Name	L*	a*	B*	ΔE
Convection drying 60 °C	53.18 ± 0.46	7.63 ± 0.2	19.76 ± 0.45	57.24

**Table 9:** True density of apple pomace dried by convection at 60 °C.

Sample Name	m [g]	V [cm^3^]	True Density [kg/m^3^]
Convection drying 60 °C	1.559	1.111	1403.474

**Table 10:** Total polyphenol content and antioxidant activity of convection-dried apple pomace at 60 °C tested by ABTS and FRAP methods.

Average Polyphenols [mg KG/100 g]	750.060
Average FRAP [µmol TE/100 g product]	805.595
Average ABTS [µmol Trolox/100 g]	624.985

## Data Availability

Not applicable.
